# Graded multisensory neurofeedback via the iNems system for comprehensive upper limb recovery after stroke: a multiple baseline study

**DOI:** 10.3389/fnhum.2026.1938622

**Published:** 2026-10-07

**Authors:** Takayuki Kodama, Ken Kitai, Yasuhisa Mizomoto, Kaichi Nishigaya, Shoutarou Hashimoto, Riona Hiraga, Satomi Shimizu, Miki Kawabata, Yuumi Hirabayashi, Keita Ikesue, Hazime Kako, Syouta Yasui, Hiroki Ito, Kazuhei Nishimoto, Osamu Katayama, Hideaki Yamaguchi, Hikaru Nagano, Mari Inoue, Shin Murata, Kiichiro Morita

**Affiliations:** 1 Graduate School of Health Sciences, Kyoto Tachibana University, Kyoto, Japan; 2 Visiting Researcher, Kyoto Tachibana University, Kyoto, Japan; 3 Watanabe Hospital, Zuisinkai Medical Corporation, Mihama, Aichi, Japan; 4 Zenjokai Rehabilitation Hospital, Zenjokai Medical Foundation, Nagoya, Aichi, Japan; 5 Sun Baden, Geriatric Health Services Facility, Mihama, Aichi, Japan; 6 National Center for Geriatrics and Gerontology, Obu, Aichi, Japan; 7 CARETECH Plus Inc., Aichi, Nagoya, Japan; 8 Faculty of Fiber Science and Engineering, Kyoto Institute of Technology, Kyoto, Japan; 9 Graduate School of Human Development and Environment, Kobe University, Kobe, Hyogo, Japan; 10 Research Institute for Brain Diseases, Kurume University, Fukuoka, Japan

**Keywords:** stroke, brain-computer interfaces, neurofeedback, iNems, upper extremity, neurorehabilitation, multisensory integration, multiple baseline design

## Abstract

**Introduction:**

Post-stroke upper-limb recovery remains challenging, especially in the presence of sensory impairments. Conventional brain–computer interfaces (BCIs) are constrained by binary feedback, single-modality stimulation, and exclusion of patients with sensory deficits. We developed the Imagery Neurofeedback-based Multi-sensory System (iNems), a neurofeedback system that provides graded multisensory (visual and electrical) feedback based on ERD magnitude. This study validated iNems’ efficacy in motor function, self-reported numbness, and cognitive functions using a multiple-baseline design (MBD).

**Methods:**

Four participants with post-stroke upper-limb motor and sensory impairments underwent a multiple baseline design. The baseline phase (phase A) used random feedback; the intervention (phase B) used adaptive iNems. iNems quantified motor intention via sensorimotor EEG event-related desynchronization (ERD) and delivered graded visual and electrical feedback. Primary outcomes were the Fugl-Meyer Assessment-Upper Extremity (FMA-UE) and Box and Block Test (BBT). Secondary outcomes included body cognition, numbness, and neurophysiological (ERD/ERS) changes.

**Results:**

All participants demonstrated significant improvement during the intervention. FMA-UE increased by 6–16 points. The BBT showed large effects (integrated Tau-U = 0.75). Body cognition improved significantly in both sense of agency (Tau-U = 0.80) and ownership (Tau-U = 0.89). Numbness was reduced in affected cases (Tau-U = −0.85). Neurophysiological changes correlated with functional gains, with some patterns suggesting increased neural efficiency.

**Conclusion:**

This first MBD-validated demonstration shows that graded, multisensory graded neurofeedback based on ERD magnitude can improve motor, self-reported numbness, and cognitive domains after stroke. These findings support adaptive closed-loop therapy as a promising, multifaceted rehabilitation strategy.

## 1 Introduction

Stroke is a leading cause of long-term disability and mortality worldwide, imposing an immense social and economic burden (GBD 2019 Stroke collaborators, 2021; Virani et al., 2021; Owolabi et al., 2022). With aging populations, the number of stroke cases is projected to increase significantly, exacerbating this challenge (Wafa et al., 2020). The economic burden of stroke extends beyond direct healthcare expenditures to include substantial indirect costs resulting from productivity losses, particularly among working-age individuals (Rajsic et al., 2019; Rochmah et al., 2021). Therefore, the development of effective post-stroke therapeutic strategies is a critical global health priority.

Among the many sequelae of stroke, upper limb motor dysfunction is one of the most common and debilitating, affecting approximately 65% of patients in the acute phase and directly impacting activities of daily living (ADL) (Parker et al., 1986; Nakayama et al., 1994). The prognosis for functional recovery remains poor for many survivors. A systematic review revealed that approximately 65% of patients continue to experience some form of upper-limb dysfunction three months post-stroke, with fine motor control of the fingers being particularly difficult to restore (Coupar et al., 2012). While a “critical period” for neuroplasticity exists within the first 5 months, the probability of regaining functional dexterity is alarmingly low for those with severe initial paralysis (Jørgensen et al., 1995; Kwakkel et al., 2003). At 1 year post-stroke, only a fraction of patients achieve practical use of their affected arm, highlighting a significant unmet need in rehabilitation (Winters et al., 2016; Kwakkel et al., 2019). This persistent dysfunction is strongly associated with diminished ADL independence and a reduced quality of life (QOL), impacting everything from basic self-care to social participation and vocational activities (Wyller et al., 1997; Lai et al., 2002; Nichols-Larsen et al., 2005).

Current rehabilitation paradigms are largely founded on Hebbian principles—“neurons that fire together, wire together”—and promote neuroplasticity through use-dependent, task-oriented training (Hebb, 1949; Kleim and Jones, 2008). This has led to the development of various effective interventions, such as Constraint-Induced Movement Therapy (Taub et al., 2006; Wolf et al., 2006), Functional Electrical Stimulation (FES) (Howlett et al., 2015), Repetitive Transcranial Magnetic Stimulation (rTMS) (Hsu et al., 2012), and robot-assisted training (Mehrholz et al., 2018). While these methods have demonstrated consistent benefits, they face several fundamental limitations.

First, while active movement therapies naturally provide sensorimotor feedback, interventions for patients with severely limited overt movement often lack the crucial temporal consistency between motor intention and resulting sensory feedback (Kitago and Krakauer, 2013). Modern motor control theories, such as the internal model theory, posit that motor learning is driven by minimizing sensory prediction errors between an efference copy and actual sensory feedback (Miall and Wolpert, 1996; Blakemore et al., 1998; Wolpert et al., 2011).

Second, the efficacy of these interventions is often limited in patients with concomitant sensory impairments, a condition known as post-stroke sensory impairment (PSSI), which affects 50–80% of stroke survivors (Connell et al., 2008; Kessner et al., 2019). Somatosensory deficits are a major barrier to motor recovery, yet interventions targeting sensory impairment remain significantly under-researched in conventional motor rehabilitation trials, leaving a critical gap in treating this population (Schabrun and Hillier, 2009; Doyle et al., 2010).

Third, conventional rehabilitation has paid insufficient attention to disorders of body cognition, such as the sense of agency (the feeling of controlling one’s own actions) and the sense of ownership (the feeling that one’s limb belongs to oneself) (Razmus, 2017; Tsakiris, 2017). Following a stroke, these senses can become profoundly disrupted, leading to feelings of limb disownment and alienation, which correlate with poorer functional outcomes (Baier and Karnath, 2008). Restoring these cognitive functions is believed to be critical for meaningful motor recovery.

In recent years, Brain–Computer Interface (BCI) technology has emerged as a promising tool to overcome these limitations (Monge-Pereira et al., 2017; Cervera et al., 2018). BCIs can establish a direct communication pathway from the brain to an external device, detecting motor intentions from neural activity in real-time and providing synchronized feedback (Ramos-Murguialday et al., 2013). This forms a “closed-loop” neuromodulation system that can selectively reinforce specific neural circuits, promoting targeted neuroplasticity (Nishimura et al., 2013; Silversmith et al., 2021). However, the efficacy of existing BCI systems has been variable, with meta-analyses reporting moderate effect sizes (Mansour et al., 2022). This variability may stem from several design limitations. Recent robust randomized controlled trials have demonstrated the significant efficacy of BCI training using motor imagery-contingent multisensory feedback (visual and functional electrical stimulation) in both subacute (Kim et al., 2024) and chronic stroke (Kim et al., 2025) populations. Recent literature has extensively investigated advanced user training and feedback methods in neurotechnology (Lotte et al., 2013; Roc et al., 2021; Mladenović, 2021). Building upon these advancements, our study aims to translate these concepts into a clinical setting. While some advanced systems successfully integrate multisensory inputs (Kim et al., 2024; Kim et al., 2025), many conventional clinical neurofeedback paradigms still rely on binary (“on/off”) triggers rather than graded mapping (Cervera et al., 2018; Mansour et al., 2022). Electrophysiological studies have shown that motor intention is not binary but is encoded as a graded neural signal, reflected in the degree of event-related desynchronization (ERD) of sensorimotor rhythms (Pfurtscheller and Lopes da Silva, 1999). Furthermore, the brain’s motor system is inherently multisensory, integrating visual, somatosensory, and auditory information to create coherent representations of the body and movement (Stein and Stanford, 2008; Blanke, 2012).

To address these shortcomings, we built upon a pioneering system known as the Imagery Neurofeedback-based Multi-sensory System (iNems), which synchronized visual and electrical feedback with detected motor intention (Kodama et al., 2019). While a case study showed promising results, the original system was limited to a single case of brainstem stroke, did not provide graded feedback, and did not evaluate effects on body cognition. We therefore developed an improved iNems system with three key innovations: (1) it quantifies the ERD magnitude and classifies it into three discrete levels; (2) it delivers graded visual and electrical feedback proportional to this detected intensity, promoting natural multisensory integration; and (3) it uses an individualized thresholding process to optimize the intervention for each patient. This approach is grounded in predictive coding theory, which proposes that the brain updates its internal models by minimizing prediction errors (Friston, 2010). By precisely aligning sensory feedback (the actual input) with motor intention (the prediction), iNems aims to facilitate efficient updating of these internal models. Therefore, the primary novelty of this study lies not in complex machine-learning classification, but in the clinical integration of graded multisensory feedback tailored to the ERD magnitude. Although the system is named the Imagery Neurofeedback-based Multi-sensory System (iNems), we explicitly define its mechanism in this study as biosignal-based multisensory feedback (i.e., presenting real-time stimuli triggered by sensorimotor rhythms), rather than traditional neurofeedback aimed at normalizing abnormal EEG patterns.

This study aimed to systematically evaluate the efficacy of the enhanced iNems system using a multiple-baseline design (MBD) across participants (Kazdin, 2011). We hypothesized that graded, multisensory feedback training tailored to the ERD magnitude would demonstrate superior efficacy in improving upper limb motor function, reducing self-reported numbness, and enhancing body cognition in a diverse group of stroke patients.

## 2 Methods

### 2.1 Study design

This study employed a multiple baseline single-case experimental design (SCED) across participants, adhering to the Single-Case Reporting Guideline in Behavioral Interventions (SCRIBE) 2016 statement (Tate et al., 2016). The MBD was selected to explore the preliminary effects of the intervention while attempting to control for the intervention and observed outcomes by systematically introducing the intervention at different time points for each participant, thereby controlling for threats to internal validity such as maturation or spontaneous recovery (Baer et al., 1968; Kratochwill et al., 2013). This design allows for rigorous scientific evaluation while accounting for the inherent heterogeneity of stroke populations (Horner et al., 2005). To enhance internal validity, the baseline period (phase A) was randomly assigned to be 5, 6, 7, or 10 days for the four participants before the introduction of the intervention period (phase B) (Figure 1). To ensure objectivity, the evaluators conducting the outcome assessments were blinded to the phase in which each participant was enrolled. All intervention procedures were manualized to ensure consistency across therapists.

**Figure 1:**
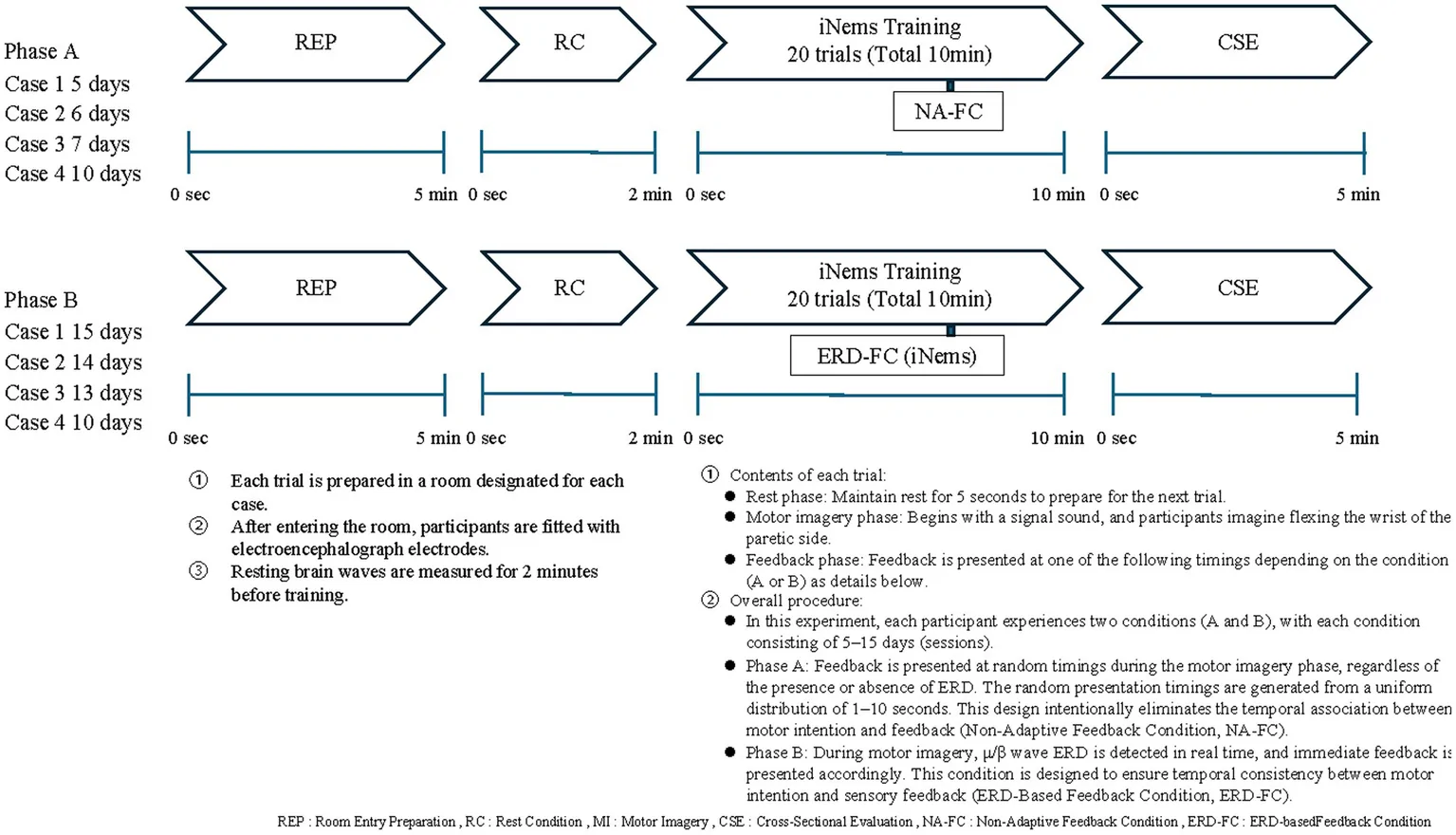
Experimental procedure. Timeline of a single experimental session, including preparation (REP), rest condition (RC), iNems training block (20 sets of 15 s), and cross-sectional evaluation (CSE). Feedback condition for phase A (non-adaptive feedback condition, NA-FC) and Phase B (ERD-based feedback condition, ERD-FC) is differentiated.

### 2.2 Participants

Participants were recruited between October 2024 and March 2025 from the inpatient rehabilitation wards of two hospitals (A and B). The inclusion criteria were as follows: (1) diagnosis of a first-ever ischemic or hemorrhagic stroke; (2) time since onset between 1 and 6 months (recovery phase); (3) presence of upper limb motor impairment (Fugl-Meyer Assessment-Upper Extremity [FMA-UE] score between 0 and 66); (4) presence of sensory impairment or abnormal sensation in the fingers; (5) age 18 years or older; and (6) ability to provide written informed consent. Exclusion criteria included the following: (1) severe cognitive impairment (Mini-Mental State Examination [MMSE] score ≤ 23); (2) severe aphasia or apraxia that would interfere with understanding instructions; (3) contraindications to electrical stimulation (e.g., pacemaker); and (4) a history of other major neurological or psychiatric disorders.

Four participants (mean age 56.8 ± 22.4 years; two males, two females) who satisfied all criteria were enrolled. All participants maintained adequate cognitive function (mean MMSE 29.75 ± 0.43). Detailed clinical characteristics of each participant are provided in Table 1.

**Table 1:** Characteristics of participants.

Case	I	II	III	IV
Sex, Age	Female, 60	Female, 88	Male, 44	Male, 35
Height (cm)	161	145	171	166
Weight (kg)	50.1	42.0	64.7	71.9
BMI	19.3	19.8	25.1	26.1
Diagnosis	Right subcortical hemorrhage	Cardiogenic cerebral infarction	Multiple cerebral infarctions	Left putaminal hemorrhage
Paretic hand	Left	Left	Right	Right
Fugl-Meyer assessment: upper extremity (points)	49	4	4	48
Severity of motor paralysis	Mild	Severe	Severe	Mild
Finger numbness (NRS)	3	10	0	6
Tactile sensation of fingers	Decreased	Decreased	Decreased	Decreased
Kinesthetic sensation of fingers (out of 10)	1	5	8	7
Time since onset at study start	~5 months	~4 months	~2 months	~3 months
Medical history	None	Atrial fibrillation, hypertension, dyslipidemia	Post-op aortic stenosis, Post-op ventricular septal defect, Hypertension	Hypertension

### 2.3 Intervention: the iNems system

The iNems system is a non-invasive, EEG-based neurofeedback system designed to provide real-time, graded, multisensory feedback (Figure 2). Hardware: The system comprised an 8-channel wireless EEG system (OpenBCI) for recording brain activity, an electrical stimulation device (Burning Shaper BR-5100, Hirose Electric Co., Ltd., Japan) to provide somatosensory feedback to the wrist extensor muscles, and an LCD monitor for visual feedback. EEG electrodes were placed over the sensorimotor cortex (C3 or C4, depending on the lesion side) according to the international 10–20 system (Jasper, 1958). During the daily calibration phase, a 2-min resting-state baseline is recorded. Standard frequency bands (μ: 8–13 Hz, β: 13–20 Hz) were applied uniformly across participants, rather than utilizing Individual Alpha Frequency peaks. The system calculates the natural logarithm of the μ/β power ratio (log[μ/β]). While motor imagery typically induces ERD in both bands, this ratio was theoretically selected based on the hypothesis that β-band activity reflects the individual’s baseline sensorimotor state, or “status quo” (Engel and Fries, 2010). Given the high inter-subject variability in default cortical excitability (Blankertz et al., 2010), relying solely on absolute μ-band ERD may fail to accurately capture intention intensity across different patients. Therefore, evaluating μ-rhythm desynchronization relative to the β baseline serves as a necessary normalization mechanism. This ratio was utilized purely as an empirical control feature to practically account for each participant’s unique default neural activity level within this specific clinical setup. Based on the maximum trial-averaged 1.0-s window peak ERD achieved during calibration, individualized thresholds were adapted daily: Threshold 1 (20–40% of max ERD), Threshold 2 (40–60%), and Threshold 3 (>60%).

**Figure 2:**
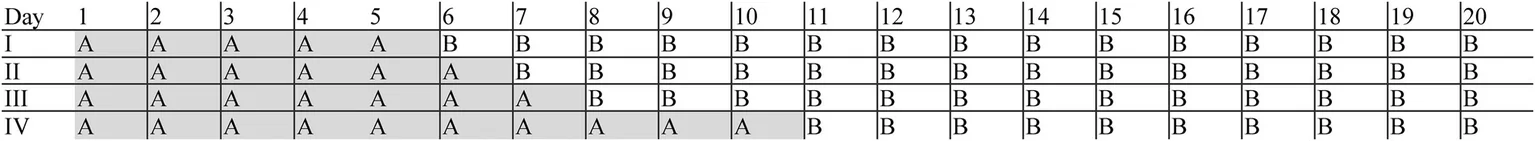
Multilevel baseline schedule between participants I–IV. Staggered introduction of the intervention (Phase B, white cells) following the baseline period (Phase A, grey cells) for each of the four participants over 20 days.

Software and Feedback Mechanism: Unlike typical BCI systems that rely on complex machine learning algorithms for automated classification, our custom iNems system utilizes a direct, individualized threshold-based discrete, three-level graded feedback approach. This design choice prioritizes real-time, proportional multisensory feedback over binary classification. Custom software analyzed EEG signals in real-time to quantify motor intention. This was achieved by calculating the ERD, a decrease in power in the μ (8–13 Hz) and β (13–20 Hz) frequency bands, which is a well-established marker of motor cortex activation (Neuper et al., 2009). For online processing, the EEG data acquired by the OpenBCI system at 200 Hz were transmitted to the host PC via serial communication. The custom software (developed in Delphi) performed real-time frequency analysis utilizing a 1.0-s sliding window, updating continuously every 125 ms to compute the ERD. Prior to spectral estimation, the raw EEG data were bandpass filtered (4–30 Hz) to remove baseline drift and high-frequency noise. To mitigate noise during online classification, epochs containing signals exceeding an amplitude threshold of ±100 μV were automatically discarded to prevent blink or muscle artifacts from triggering the system. The software evaluated the threshold conditions dynamically and rendered the visual feedback directly to the LCD monitor, simultaneously streaming execution triggers to the FES device via an M5 microcontroller (programmed via Arduino) using serial communication. This direct internal pipeline enabled the system to synchronize intention detection with multisensory feedback. The system calculated the natural logarithm of the μ/β power ratio (log[μ/β]) as a stable indicator of motor intention. Based on the decay rate of this indicator relative to a resting baseline, the ERD magnitude was classified into three levels, with individualized thresholds set during a calibration session. The system then delivered proportional multisensory feedback:

- Threshold 1 (Weak Intention; 20–40% ERD): A video of the wrist flexing 30°, combined with electrical stimulation at the sensory threshold (perceptible, no muscle contraction) for 2 s.
- Threshold 2 (Moderate Intention; 40–60% ERD): A video of the wrist flexing 60°, combined with electrical stimulation at the motor threshold (mild muscle contraction) for 3 s.
- Threshold 3 (Strong Intention; >60% ERD): A video of the wrist in maximum dorsiflexion, combined with functional electrical stimulation (clear joint movement) for 4 s.

### 2.4 Experimental protocol

The protocol was approved by a certified Institutional Review Board, and all patients provided signed informed consent prior to participation. All participants continued their standard rehabilitation program throughout the study. The iNems intervention was added for one session per day, five days a week, totaling 20 sessions per participant. To prevent cognitive fatigue and maintain the high quality of motor imagery—factors critical for post-stroke populations—the session length was strictly limited to 20 min. This duration was selected because motor imagery effectiveness has been shown to peak at approximately 20 min before declining due to mental fatigue (Driskell et al., 1994), which aligns with established clinical best practices for mental training (Schuster et al., 2011).

EEG data was acquired using an OpenBCI system at a sampling frequency of 200 Hz. Conductive paste-based electrodes were utilized, with impedance strictly maintained below 5 kΩ. The reference and ground electrodes were placed on the right earlobe and FPz, respectively.

Phase A (active non-contingent-feedback control) and Phase B (intervention) were conducted strictly sequentially. Each daily session commenced with the aforementioned calibration phase. During the trials, participants were seated comfortably and performed 20 trials of motor imagery (MI) of wrist dorsiflexion versus a resting condition, guided by machine-instructed visual cues (Figure 3). An inter-trial pause of 5 s was provided between each motor imagery trial to prevent cognitive and physiological fatigue.

- Phase A (active non-contingent-feedback control): Participants performed the motor imagery task, but the visual and electrical feedback was delivered at random intervals, independent of their brain activity. This controlled for the non-contingent effects of stimulation and motor practice.
- Phase B (Intervention): Feedback was delivered contingently and was graded according to the real-time ERD magnitude, as described above. The custom software dynamically evaluated these threshold conditions, seamlessly switching the corresponding visual and electrical feedback states in real-time.

**Figure 3:**
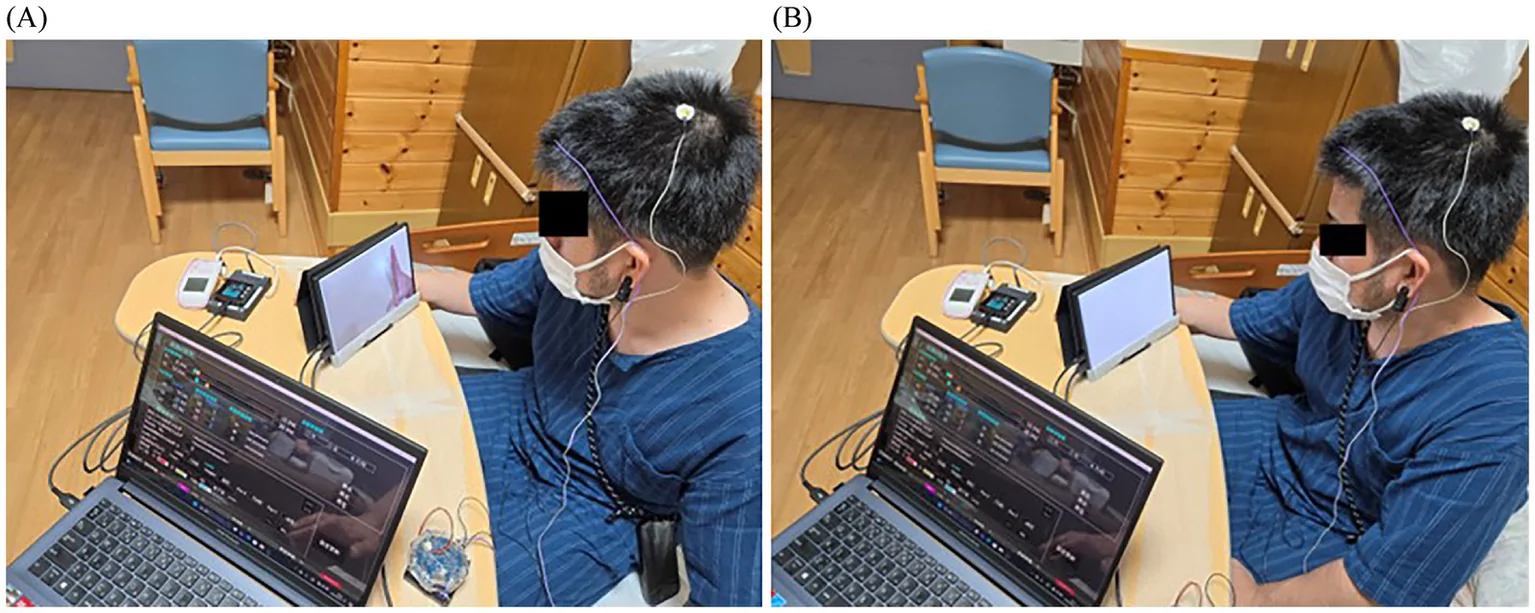
Training scene using imagery Neurofeedback-based Multi-sensory Systems (iNems). **(A)** A participant engages in iNems training; when motor imagery is successful, the hand on the screen moves. **(B)** The screen before successful motor imagery; during training, successful motor imagery, detected as ERD, triggers synchronized visual feedback (finger movement on screen) and electrical stimulation to the corresponding muscles.

### 2.5 Outcome measures

A comprehensive set of measures was used to evaluate outcomes from multiple perspectives. Longitudinal Evaluation (Daily Measures):

For longitudinal evaluations, the Box and Block Test (BBT) and Numerical Rating Scale (NRS) for sense of agency, sense of ownership, and numbness were conducted daily immediately prior to the start of each iNems training session. Average values were not utilized for these indices; rather, the actual raw values measured in each daily session were used directly as the results for the longitudinal single-case analysis.

- Box and Block Test (BBT): To assess changes in manual dexterity, this standardized test measured the number of blocks transferred in 60 s (Mathiowetz et al., 1985).
- Numerical Rating Scale (NRS): An 11-point scale (0–10) was used to rate the intensity of numbness and two aspects of body cognition: sense of agency (“I feel I am controlling my arm movements”) and sense of ownership (“My arm feels like it belongs to me”) (Hawker et al., 2011).

Cross-sectional evaluation (pre- and post-intervention):

- Fugl-Meyer Assessment-Upper Extremity (FMA-UE): The gold standard for measuring post-stroke motor impairment, scored on a 66-point scale (Fugl-Meyer et al., 1975).
- Motor Activity Log-14 (MAL-14): A self-report questionnaire assessing the perceived amount of use (AOU) and quality of movement (QOM) of the paretic arm in daily life (Uswatte et al., 2006).
- Psychological Scales: The Pain Catastrophizing Scale (PCS) (Sullivan et al., 1995) and the Hospital Anxiety and Depression Scale (HADS) (Zigmond and Snaith, 1983) were used to assess psychological factors.

Neurophysiological evaluation:

- EEG data recorded during each session were analyzed offline to quantify changes in ERD (pre-feedback μ-wave power) and ERS (post-feedback β-wave power). ERS, a rebound in power, is believed to reflect feedback processing and learning consolidation.

For cross-sectional evaluations, the MAL-14 (AOU and QOM) is scored on a 0 to 5 scale, with higher scores indicating better perceived arm use and movement quality. The PCS assesses pain catastrophizing on a scale of 0–52, where higher scores reflect greater psychological distress. The HADS evaluates anxiety and depression on a scale of 0–21 for each subscale, with higher scores indicating more severe symptoms.

### 2.6 Data analysis

For longitudinal data (BBT, NRS), visual analysis of graphs was conducted to assess changes in level, trend, and variability between phases. Statistical analysis was conducted using the Tau-U method, a nonparametric approach that estimates an effect size by combining the degree of non-overlap and the trend between phases, while correcting for any baseline trend (Parker and Vannest, 2009; Parker et al., 2011). For cross-sectional data, changes in measurement points (start of A, start of B, end of B) were visually analyzed. Neurophysiological data (ERD/ERS) were compared between phase A and phase B to evaluate intervention-induced changes. Statistical differences in mean ERD and ERS power between the two phases were determined using Welch’s t-test to account for unequal variances and sample sizes across trials. EEG data analysis was performed using the EEGLAB toolbox in MATLAB R2026a (The MathWorks, Inc., Natick, MA, USA) (Delorme and Makeig, 2004). Continuous EEG data were segmented into epochs from −3.0 to 6.0 s relative to the visual cue onset to isolate the specific motor imagery periods. Furthermore, time-frequency distributions were computed using continuous wavelet transforms to visually evaluate the dynamic changes in relative log spectral power (dB). Based on the experimental design, specific regions of interest (ROIs) were defined. For ERD (μ-band: 8–13 Hz), the analysis window was set to 1.0 s immediately preceding the feedback trigger (2.0–3.0 s of the epoch) to capture the peak ERD magnitude. For ERS (β-band: 13–20 Hz), the window was set to 3.0 s immediately following the trigger (3.0–6.0 s). This 3.0-s post-trigger window optimally covers the duration of the graded multisensory feedback (2–4 s), allowing for the visual assessment of sensory feedback processing and subsequent cortical rebound. All statistical analyses were conducted using IBM SPSS Statistics version 27.0 (IBM Corp., Armonk, NY, USA). Given the single-case design, comparing trial-level data via Welch’s t-test involves repeated measures that violate the assumption of independence (pseudoreplication). Therefore, these specific neurophysiological statistical results are presented strictly as exploratory and descriptive rather than robust inferential evidence.

## 3 Results

### 3.1 Study completion and safety

All four participants successfully completed the study protocol without any adverse events.

### 3.2 Motor function improvement

The intervention resulted in significant improvements in motor function for all participants.

FMA-UE: FMA-UE: All cases demonstrated an increase in total FMA-UE scores during phase B (Table 2). The gains ranged from +6 to +16 points. Case I (mild paralysis) improved from 50 to 56 points, and Case IV (mild paralysis) improved from 48 to 56 points. Notably, the participants with severe initial paralysis demonstrated substantial gains: Case II improved from 6 to 12 points, and Case III improved from 32 to 48 points. Improvements were observed in both proximal (shoulder/elbow) and distal (finger) subscales, as well as in coordination.

**Table 2:** FMA-UE.

Case / Phase	Scores
I	
Initial	Total: 49/66 Shoulder/Elbow/Forearm: 28/36, Wrist 6/10, Fingers 10/14, Coordination/Speed 05/06
A	Total: 50/66 Shoulder/Elbow/Forearm: 28/36, Wrist 6/10, Fingers 11/14, Coordination/Speed 05/06
B	Total: 56/66 Shoulder/Elbow/Forearm: 34/36, Wrist 6/10, Fingers 11/14, Coordination/Speed 05/06
II	
Initial	Total: 04/66 Shoulder/Elbow/Forearm: 04/36, Wrist 00/10, Fingers 00/14, Coordination/Speed 00/06
A	Total: 06/66 Shoulder/Elbow/Forearm: 06/36, Wrist 00/10, Fingers 00/14, Coordination/Speed 00/06
B	Total: 12/66 Shoulder/Elbow/Forearm: 12/36, Wrist 00/10, Fingers 00/14, Coordination/Speed 00/06
III	
Initial	Total: 04/66 Shoulder/Elbow/Forearm: 04/36, Wrist 00/10, Fingers 00/14, Coordination/Speed 00/06
A	Total: 32/66 Shoulder/Elbow/Forearm: 16/36, Wrist 05/10, Fingers 11/14, Coordination/Speed 00/06
B	Total: 48/66 Shoulder/Elbow/Forearm: 24/36, Wrist 06/10, Fingers 14/14, Coordination/Speed 04/06
IV	
Initial	Total: 48/66 Shoulder/Elbow/Forearm: 25/36, Wrist 09/10, Fingers 14/14, Coordination/Speed 00/06
A	Total: 48/66 Shoulder/Elbow/Forearm: 25/36, Wrist 09/10, Fingers 14/14, Coordination/Speed 00/06
B	Total: 56/66 Shoulder/Elbow/Forearm: 31/36, Wrist 08/10, Fingers 14/14, Coordination/Speed 03/06

FMA, Fugl-Meyer assessment; UE, upper extremity.

BBT: For manual dexterity, three of the four participants showed statistically significant improvements after the intervention began (Table 3).

**Table 3:** BBT.

Case	Phase A AVG ± SE	Phase B AVG ± SE	Tau-U	VARs	SD	Z	*p* value	90% CI
I	21.40 ± 1.47	26.07 ± 0.52	0.88	525	22.91	2.79	0.0052	0.351< >1.000
II	00.00 ± 0.00	00.00 ± 0.00	0.00	588	24.25	0.00	1.0000	−0.475< >0.475
III	09.86 ± 0.63	17.00 ± 0.93	0.99	637	25.24	3.53	0.0004	0.522< >1.000
IV	17.40 ± 0.83	22.50 ± 1.26	0.75	700	26.46	2.76	0.0058	0.295< >1.000
Combine			0.75					

BBT, Box and Block Test.

Case I improved from a baseline mean of 21.4 blocks to an intervention mean of 26.1 blocks (Tau-U = 0.88, *p* = 0.005).

Case III revealed the largest improvement, from 9.9 blocks to 17.0 blocks (Tau-U = 0.99, *p* < 0.001).

Case IV improved from 17.4 blocks to 22.5 blocks (Tau-U = 0.75, *p* = 0.006).

Case II, who presented with the most severe paralysis, was unable to transfer any blocks throughout the study. The integrated Tau-U across all four cases was 0.75, indicating a large overall effect size.

### 3.3 Improvement in body cognition and sensory impairment

The intervention led to marked improvements in both body cognition and sensory perception. Body Cognition: A sense of agency and ownership improved significantly (Table 4).

**Table 4:** Body cognition.

Case	Phase A AVG ± SE	Phase B AVG ± SE	Tau-U	VARs	SD	Z	*p* value	90% CI
Sense of agency								
I	04.00 ± 0.00	05.07 ± 0.23	0.80	525	22.91	2.18	0.0291	0.164< >1
II	06.00 ± 1.90	09.00 ± 0.00	0.50	588	24.25	1.15	0.2482	−0.142< >0.808
III	05.43 ± 0.20	07.31 ± 0.26	0.89	637	25.24	3.09	0.0020	0.401< >1
IV	03.10 ± 0.18	05.40 ± 0.48	0.90	700	26.46	3.21	0.0013	0.415< >1
Combine			0.80					
Sense of ownership								
I	08.20 ± 0.20	08.93 ± 0.12	0.78	525	22.91	2.23	0.0260	0.177< >1
II	05.67 ± 1.80	09.07 ± 0.07	0.82	588	24.25	2.39	0.0168	0.216< >1
III	05.00 ± 0.00	07.31 ± 0.26	0.96	637	25.24	3.33	0.0009	0.467< >1
IV	01.80 ± 0.29	04.00 ± 0.30	0.94	700	26.46	3.33	0.0009	0.445< >1
Combine			0.89					

Sense of Agency: Significant improvements were observed in three of four cases, with Tau-U values ranging from 0.80 to 0.90. The integrated Tau-U was 0.80, a very large effect.

Sense of Ownership: All four participants showed significant improvements, with Tau-U values ranging from 0.78 to 0.96. The integrated Tau-U was 0.89, also a very large effect.

Numbness: All three participants who reported numbness at baseline experienced a significant reduction during phase B (Table 5).

**Table 5:** NRS for numbness.

Case	Phase A AVG ± SE	Phase B AVG ± SE	Tau-U	VARs	SD	Z	*p* value	90% CI
I	03.00 ± 0.00	02.00 ± 0.20	−0.8462	525	22.91	−2.40	0.0164	−1.000< > − 0.231
II	10.00 ± 0.00	09.07 ± 0.12	−0.9231	588	24.2487	−2.97	0.0030	−1.000< > − 0.382
III	00.00 ± 0.00	00.00 ± 0.00	0.00	637	25.24	0.00	1.0000	−0.456< > 0.456
IV	04.00 ± 0.00	02.80 ± 0.25	−0.8889	700	26.46	−3.02	0.0025	−1.000< > − 0.365
Combine			−0.7433					

NRS, numerical rating scale.

Significant reductions in NRS scores were observed across all cases. In Case I, the mean NRS score decreased from 3.0 to 2.0 (Tau-U = −0.85, *p* = 0.016). In Case II, the mean score declined from 10.0 to 9.07 (Tau-U = −0.92, *p* = 0.003). Similarly, Case IV demonstrated a reduction in mean NRS score from 4.0 to 2.8 (Tau-U = −0.89, *p* = 0.003).

The integrated Tau-U for these three cases was −0.85, indicating a very large therapeutic effect on this challenging sensory symptom.

### 3.4 Changes in ADL and psychological state

MAL-14: Improvements in motor function translated to increased use of the paretic arm in daily life for three participants (Table 6). Case III showed a particularly notable increase in both AOU and Quality of Movement (QOM) scores.

**Table 6:** MAL-14.

Case / Phase	AoU	QoM
I		
Initial	3.70	3.85
A	3.70	3.85
B	4.50	4.60
II		
Initial	0.00	0.00
A	0.00	0.00
B	0.00	0.00
III		
Initial	0.85	0.92
A	1.31	1.31
B	2.54	2.54
IV		
Initial	1.9	1.4
A	1.9	1.4
B	2.2	1.9

MAL-14, Motor Activity Log-14.

Psychological Outcomes: There was a trend toward improved psychological well-being. PCS scores, reflecting catastrophic thinking about pain/numbness, decreased in all cases during phase B (Table 7). HADS scores also demonstrated improvements in anxiety for three cases and in depression for two cases (Table 8).

**Table 7:** PCS.

Case / Phase	Total and subscale scores
I	
Initial	Total: 10/52 Rumination: 6/20, Helplessness: 4/20, Magnification: 0/12
A	Total: 10/52 Rumination: 6/20, Helplessness: 4/20, Magnification: 0/12
B	Total: 8/52 Rumination: 6/20, Helplessness: 2/20, Magnification: 0/12
II	
Initial	Total: 44/52 Rumination: 20/20, Helplessness: 16/20, Magnification: 8/12
A	Total: 39/52 Rumination: 17/20, Helplessness: 15/20, Magnification: 7/12
B	Total: 23/52 Rumination: 13/20, Helplessness: 4/20, Magnification: 6/12
III	
Initial	Total: 11/52 Rumination: 5/20, Helplessness: 2/20, Magnification: 4/12
A	Total: 11/52 Rumination: 5/20, Helplessness: 2/20, Magnification: 4/12
B	Total: 4/52 Rumination: 2/20, Helplessness: 1/20, Magnification: 1/12
IV	
Initial	Total: 19/52 Rumination: 9/20, Helplessness: 5/20, Magnification: 5/12
A	Total: 19/52 Rumination: 9/20, Helplessness: 5/20, Magnification: 5/12
B	Total: 10/52 Rumination: 6/20, Helplessness: 2/20, Magnification: 2/12

PCS, Pain Catastrophizing Scale.

**Table 8:** HADS.

Case / Phase	Anxiety	Depression
I		
Initial	3.00	0.00
A	2.00	0.00
B	1.00	0.00
II		
Initial	17.00	10.00
A	16.00	12.00
B	17.00	10.00
III		
Initial	9.00	10.00
A	9.00	10.00
B	6.00	7.00
IV		
Initial	3.00	4.00
A	1.00	3.00
B	1.00	1.00

HADS, Hospital Anxiety and Depression Scale.

### 3.5 Neurophysiological changes

EEG analysis revealed intervention-induced neuroplastic changes, although the patterns were heterogeneous (Table 9).

**Table 9:** ERD and ERS.

		ERD				ERS			
Case	Comparison	μ (8–13 Hz)		*t*-value	*p*-value	β (13–20 Hz)		*t*-value	*p*-value
		Phase A	Phase B			Phase A	Phase B		
I	AvsB (AVGμV ± SE)	5.91 ± 0.19	4.56 ± 0.10	6.21	< 0.001	7.40 ± 0.22	5.18 ± 0.19	8.85	< 0.001
II	AvsB (AVGμV ± SE)	6.93 ± 0.14	6.05 ± 0.10	5.03	< 0.001	8.12 ± 0.12	7.32 ± 0.11	4.75	< 0.001
III	AvsB (AVGμV ± SE)	3.65 ± 0.18	4.54 ± 0.10	−4.23	< 0.001	4.44 ± 0.23	6.02 ± 0.13	−5.85	< 0.001
IV	AvsB (AVGμV ± SE)	6.22 ± 0.07	6.88 ± 0.07	−6.43	< 0.001	8.17 ± 0.05	8.46 ± 0.04	−5.1	< 0.001

ERD, event-related desynchronization; ERS, event-related synchronization. Differences between phases A and B were analyzed using Welch’s t-test.

ERD (μ-wave): In Cases I and II, ERD was enhanced during phase B (power values decreased), indicating stronger activation of the motor cortex. Conversely, in Cases III and IV, which showed the most significant functional gains, ERD was attenuated (power values increased). This seemingly paradoxical finding may reflect an increase in neural efficiency, where the brain can generate motor intentions with fewer synchronized neural resources as motor control improves.

ERS (β-wave): Changes in β-wave ERS post-feedback also showed similar patterns, with reductions in power for Cases I and II and increases for Cases III and IV. These changes indicate that the intervention altered not only the generation of motor commands but also the processing of sensory feedback and the consolidation of motor learning.

To visually elucidate these neurophysiological shifts, time-frequency heatmaps based on wavelet transforms are presented in Figure 4. The defined regions of interest—indicated by the blue boxes for μ-band ERD and red boxes for β-band ERS—directly corroborate the numerical data in Table 9, visually demonstrating the intervention-induced modulation of cortical activity before and during the multisensory feedback processing window.

**Figure 4:**
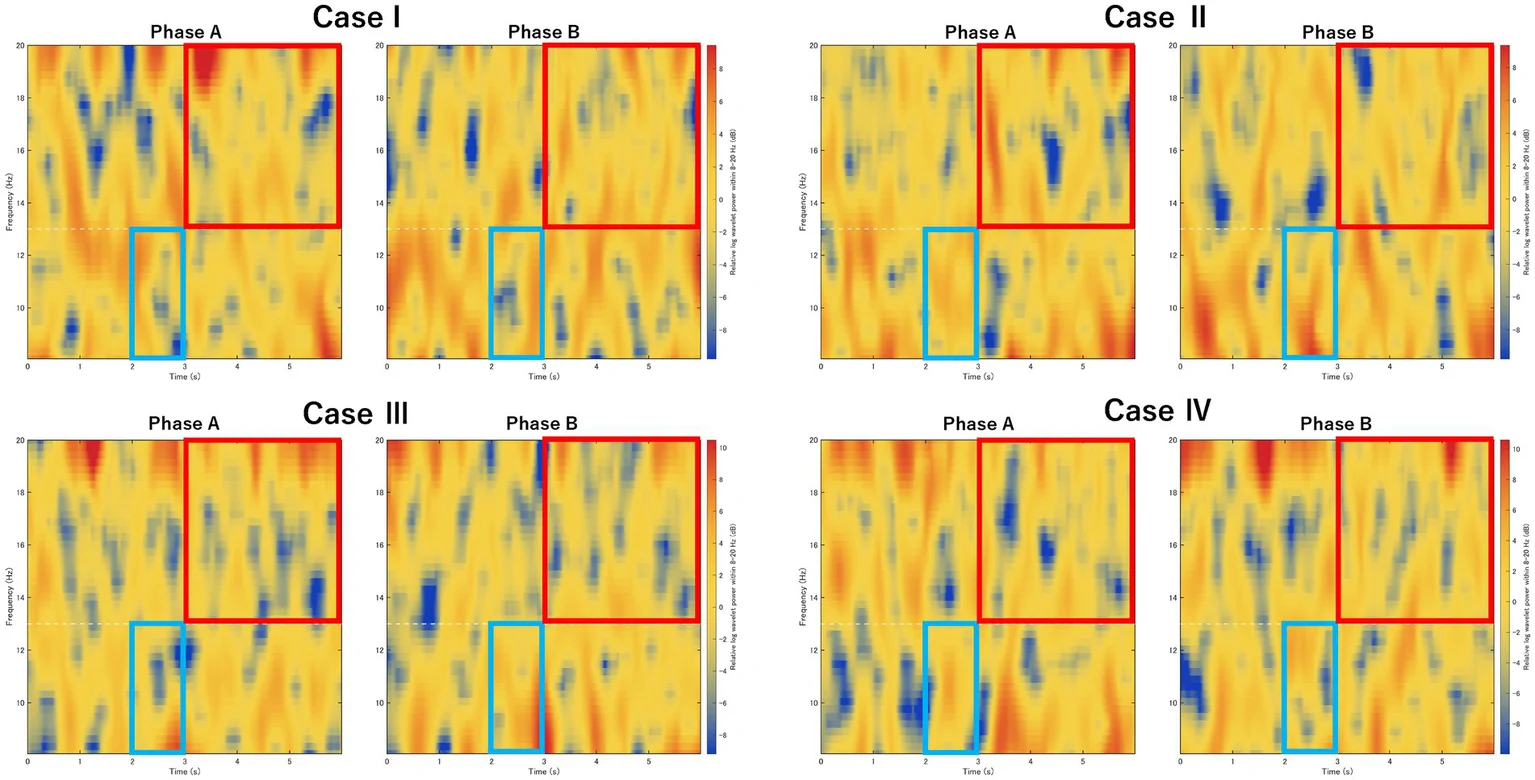
Time-frequency heatmaps of relative log spectral power derived from continuous wavelet transforms for Cases I–IV. The panels compare neurophysiological activity between Phase A (active non-contingent-feedback control) and Phase B (intervention). The *x*-axis represents time (s), with the feedback trigger occurring at 3.0 s. The *y*-axis represents frequency (Hz). The color scale indicates relative log spectral power in decibels (dB). To correspond with the numerical data in Table 9, two regions of interest (ROIs) are highlighted: the blue box denotes the μ-band event-related desynchronization (ERD) analysis window (8–13 Hz, 1.0 s prior to the trigger [2.0–3.0 s]), and the red box denotes the β-band event-related synchronization (ERS) analysis window (13–20 Hz, 3.0 s immediately following the trigger [3.0–6.0 s]). ERD, event-related desynchronization; ERS, event-related synchronization; Hz, Hertz; s, seconds; dB, decibels.

### 3.6 Online trigger rate

During the intervention phase (Phase B), the online classification performance of the iNems system was evaluated to determine how reliably participants could trigger the contingent feedback. The average Trigger Rate—defined as the percentage of cued motor imagery trials where the participant successfully crossed Threshold 1 (20–40% of maximum ERD) to activate the feedback—was 78.8% across all sessions (Case I: 82%, Case II: 71%, Case III: 78%, Case IV: 84%). This confirms that the graded feedback was delivered contingent upon the participants’ active motor intentions with a high degree of reliability. Importantly, because rest trials were not systematically evaluated for false positives in real-time, this metric represents sensitivity during MI trials rather than absolute classification accuracy.

## 4 Discussion

This study provides robust evidence, through a rigorous MBD, that the iNems system—a neurofeedback system that delivers graded multisensory feedback proportional to motor intention—is an effective intervention for improving upper-limb function in stroke patients. The results demonstrate clinically meaningful improvements in motor, sensory, and cognitive domains, indicating that this approach redefines the role of BCI from a compensatory tool to a therapeutic instrument that actively remodels neural networks.

### 4.1 Mechanisms of motor function improvement

The significant improvements in FMA-UE and BBT scores are likely attributable to a confluence of neuroscientific mechanisms. A primary hypothesized contributor is that the temporal coupling of motor-related cortical activity and afferent feedback may facilitate associative or Hebbian plasticity. By detecting ERD and providing timely feedback, iNems aims to synchronize motor cortex activation with peripheral sensory feedback, a condition known to be optimal for strengthening synaptic connections.

Critically, the graded nature of the feedback likely optimizes motor learning by generating appropriate sensory prediction errors. Motor learning theory indicates that learning is most efficient when there is a moderate discrepancy between predicted and actual sensory outcomes (Tseng et al., 2007; Herzfeld et al., 2014). By providing minimal feedback for weak intentions and strong feedback for robust intentions, iNems continuously challenges the brain’s internal model without being overwhelming or trivial, a more sophisticated approach than conventional binary feedback systems.

Third, multisensory integration likely drives the reorganization of cortical networks (Calvert, 2001). Simultaneous and congruent visual and somatosensory inputs are known to produce superadditive responses in multisensory brain regions. By facilitating compensation through alternative or spared neural pathways, this mechanism may help explain the significant improvements observed in participants with severe baseline paralysis, despite their traditionally poor prognosis (Kwakkel et al., 1996).

Furthermore, the varying lengths of Phase A reveal a potential confounding dynamic. Cases III and IV, who underwent longer Phase A periods, exhibited the most substantial gains. This suggests that prolonged exposure to non-contingent stimuli might possess a dose-dependent carry-over effect, which complicates the strict interpretation of the Phase B intervention effect.

Our findings align with a growing body of literature establishing the core therapeutic value of incorporating visual and somatosensory feedback into BCI-based upper-limb rehabilitation. For instance, compelling recent work by Kim et al. (2025) demonstrated the superiority of MI-contingent feedback over MI-independent feedback in stroke populations. However, while most conventional systems—including those described by Kim et al.—rely on binary feedback paradigms (i.e., a simple ‘on/off’ trigger when a specific threshold is met), our iNems system advances this framework by delivering graded, multisensory stimulation. By dynamically scaling both visual and electrical feedback across three individualized intensity levels based on real-time ERD amplitude, iNems generates more precise sensory prediction errors. This proportional feedback continuously challenges and updates the brain’s internal forward models, providing a more nuanced sensorimotor integration process that we hypothesize is critical for driving functional recovery.

### 4.2 Neurophysiological basis for improvement in sensory and body cognition

A striking finding of this study was the significant reduction in numbness and the profound improvement in body cognition (agency and ownership). These domains are intrinsically linked. Post-stroke sensory disturbances and disorders of body representation are believed to arise from a breakdown in the brain’s internal body schema due to mismatched sensorimotor signals (de Vignemont, 2010). The iNems intervention directly addresses this core deficit.

The improvement in numbness may be explained by three mechanisms. First, the precisely timed somatosensory (electrical) stimulation may help normalize processing in damaged thalamocortical circuits. Second, and perhaps more importantly, the intervention facilitates the relearning of the body schema. By consistently providing sensory feedback that is congruent with motor intention, iNems reduces prediction errors and allows the brain to rebuild a coherent internal model of the affected limb (Clark, 2013). The fact that improvements in body ownership and agency occurred in parallel with, or even preceded, motor gains supports the hypothesis that re-establishing an intact body representation is a foundational step for motor recovery. The sense of agency itself is generated when a match occurs between predicted and actual sensory feedback, a process iNems is explicitly designed to facilitate (Farrer et al., 2003). Third, the FES component may have activated endogenous descending pain inhibitory systems, contributing to the reduction of abnormal sensations (Ossipov et al., 2010).

### 4.3 Dynamics of neuroplasticity: efficiency and reorganization

The heterogeneous patterns of ERD changes offer fascinating insights into the underlying neuroplasticity. The enhancement of ERD in some participants (Cases I and II) likely reflects increased excitability and recruitment of the sensorimotor cortex. However, the attenuation of ERD in the participants with the greatest functional recovery (Cases III and IV) is particularly noteworthy. This suggests an increase in neural efficiency (Poldrack, 2015). As motor skills are acquired and refined, the brain learns to perform the task using fewer or less synchronized neural resources. Thus, a weaker ERD alongside better performance is a hallmark of successful motor learning. However, this remains a post-hoc hypothesis; alternative explanations include altered motor imagery strategies, fatigue, changing baseline spectral power, or the effects of daily threshold adaptation. The iNems intervention, therefore, appears not only to reactivate the motor system but also to promote its optimization.

Furthermore, changes in post-feedback β-band ERS suggest alterations in learning-related cognitive processing. β-ERS is associated with the processing of sensory feedback and the maintenance of the current sensorimotor state (Engel and Fries, 2010). The altered ERS patterns in phase B may reflect a strengthened process of introspection, in which the brain actively compares the iNems-driven feedback with its internal model, thereby consolidating learning and reinforcing the senses of agency and ownership.

### 4.4 Clinical implications, limitations, and future directions

This study has significant clinical implications. The positive results in patients with severe paralysis and sensory impairment indicate that this technology could benefit a patient population often excluded from advanced rehabilitation trials. The observed individual differences in response underscore the importance of personalized medicine. Factors such as the integrity of the residual corticospinal tract (Byblow et al., 2015), age, and psychological state likely influence outcomes and should be considered in patient selection and protocol design. Compared to other high-technology interventions such as robotics, the iNems system is relatively low-cost, potentially improving accessibility.

Crucially, this work must be interpreted as a preliminary pilot study. The small sample size (n = 4) and the inherent heterogeneity of the multiple baseline design—specifically the varying lengths of Phase A across participants—restrict the generalizability of our findings, although the MBD provides strong internal validity for these specific cases. The absence of a conventional therapy control group and a long-term follow-up period indicates that questions regarding comparative effectiveness and the maintenance of gains remain. Furthermore, relying on a single EEG channel (C3/C4) without advanced multivariate spatial filtering limits decoding accuracy. While this minimal setup was purposefully chosen to maximize clinical feasibility, affordability, and rapid setup time in typical hospital environments, it may yield lower decoding accuracy than multi-channel systems, potentially leading to occasional decoding errors or user frustration. Additionally, relying on standard frequency bands rather than Individual Alpha Frequency (IAF) peaks, alongside the use of the empirical μ/β ratio—where a larger ERD in the β band relative to the μ band could theoretically penalize performance—represents a methodological constraint. Finally, our mechanistic discussions must be cautiously bounded. Although our time-frequency wavelet analyses substantiate shifts in cortical surface activity, this surface-level EEG does not verify the activity of deeper interconnected brain networks. Because neuroimaging was not performed, discussions of network-level reorganization remain inferential, and we avoid over-interpreting these preliminary clinical outcomes as definitive evidence of broad neural system reorganization.

Several additional methodological limitations must be acknowledged. First, our reliance on a simple ±100 μV online amplitude rejection cannot definitively rule out contamination from EMG or FES-induced artifacts, which is a known constraint of single-channel setups. Second, because rest trials were not systematically evaluated for false positives in real-time, our online trigger rate represents sensitivity during MI rather than absolute classification accuracy. Third, the daily adaptation of ERD thresholds confounds the longitudinal interpretation of absolute neurophysiological control, as the neural criterion for feedback changed across sessions. Fourth, given the single-case design, comparing trial-level data via Welch’s t-test involves repeated measures that violate the assumption of independence (pseudoreplication); therefore, our neurophysiological statistical results are presented strictly as exploratory and descriptive rather than robust inferential evidence. Finally, reductions in subjective NRS scores reflect changes in self-reported numbness and do not equate to the objective recovery of somatosensory function.

Future research should focus on several key areas. Larger-scale randomized controlled trials are warranted to confirm these findings and establish iNems’s place in clinical practice guidelines. Combining iNems with neuroimaging techniques such as fMRI would provide more direct evidence of the neural mechanisms underlying recovery. Finally, the development of a more user-friendly, home-based version of the iNems system could dramatically increase the dose of therapy and improve long-term outcomes for stroke survivors.

## 5 Conclusion

This study demonstrates that iNems training produces clinically meaningful improvements in upper-limb motor function, body perception, and neurophysiological markers in stroke patients in the recovery phase. The multiple-baseline SCED suggests potential benefits of the intervention while preserving information regarding individual response patterns. A novel and significant finding of this study is that the restoration of body ownership and agency, coupled with enhanced motor cortex activation, suggests that multisensory integration addresses fundamental barriers to recovery in chronic stroke. These findings support further development and clinical implementation of iNems as a promising addition to stroke rehabilitation approaches.

## Data Availability

The datasets presented in this article are not readily available because they contains sensitive personal information derived from individual brainwave recordings. Due to privacy concerns and ethical considerations, these data are not publicly available. Requests to access the datasets should be directed to Kodama Takayuki, kodama-t@tachibana-u.ac.jp.

## Glossary

ADL: Activities of Daily Living
AOU: Amount of Use (from MAL-14)
BBT: Box and Block Test
BCI: Brain–computer Interface
CSE: Cross-Sectional Evaluation
ERD: Event-Related Desynchronization
ERS: Event-Related Synchronization
FES: Functional Electrical Stimulation
FMA-UE: Fugl-Meyer Assessment-Upper Extremity
HADS: Hospital Anxiety and Depression Scale
iNems: Imagery Neurofeedback-based Multi-sensory Systems
MAL: Motor Activity Log
MBD: Multiple Baseline Design
MI: Motor Imagery
MMSE: Mini-Mental State Examination
NRS: Numerical Rating Scale
PCS: Pain Catastrophizing Scale
PSSI: Post-stroke Sensory Impairment
QOL: Quality of Life
QOM: Quality of Movement (from MAL-14)
RC: Rest Condition
REP: Room Entry Preparation
rTMS: Repetitive Transcranial Magnetic Stimulation
